# Loneliness Among Black/African American Adults Living with HIV: Sociodemographic and Psychosocial Correlates and Implications for Adherence

**DOI:** 10.1007/s40615-023-01712-4

**Published:** 2023-07-12

**Authors:** Nipher Malika, Laura M. Bogart, Matt G. Mutchler, Kathy Goggin, David J. Klein, Sean J. Lawrence, Glenn J. Wagner

**Affiliations:** 1 RAND Corporation, 1776 Main St, Santa Monica, CA 90401 USA; 2 APLA Health & Wellness, Los Angeles, CA USA; 3 California State University Dominguez Hills, Carson, CA USA; 4 Children’s Mercy Kansas City and University of Missouri-Kansas City, School of Medicine and Pharmacy, Kansas City, MO USA

**Keywords:** Loneliness, HIV/AIDS, Black/African American, Sexual minority individuals, Social support

## Abstract

Loneliness, an emerging public health problem, is higher among people living with HIV and is associated with negative health outcomes. Black/African Americans have a high burden of HIV, and little is known about the characteristics of loneliness among Black adults living with HIV; therefore, this study sought to understand the sociodemographic and psychosocial correlates of Black adults living with HIV who are lonely and the implications of loneliness for their health outcomes. A sample of 304 Black adults living with HIV (73.8% sexual minority men) in Los Angeles County, CA, USA, completed the survey items assessing sociodemographic and psychosocial characteristics, social determinants of health, health outcomes, and loneliness. Antiretroviral therapy (ART) adherence was assessed electronically with the medication event monitoring system. Bivariate linear regressions analysis showed higher loneliness scores among those with higher levels of internalized HIV stigma, depression, unmet needs, and discrimination related to HIV serostatus, race, and sexual orientation. In addition, participants who were married or living with a partner, had stable housing, and reported receiving more social support had lower levels of loneliness. In multivariable regression models controlling for correlates of loneliness, loneliness was found to be a significant independent predictor of worse general physical health, worse general mental health, and greater depression. Loneliness was marginally associated with lower ART adherence. Findings suggest that Black adults living with HIV, who experience multiple intersectional stigmas, require targeted interventions and resources.

## Introduction

In 2019, Black/African American persons had a higher burden of HIV (42.1) of all racial/ethnic groups in the USA [1]. Among those Black/African American individuals with HIV, males accounted for 76% of HIV infections, most of which (82%) were attributed to male-to-male sexual contact [1]. Black/African Americans also have poorer HIV outcomes, including lower antiretroviral therapy (ART) adherence [2–5]. These health disparities may be related to findings showing that Black Americans have multiple negative social determinants that impact their health, such as poor access to education, employment, and health care and unstable housing [6–9].

Among Black/African Americans living with HIV, the impact of negative social determinants is compounded by intersectional stigma associated with their race [10, 11], sexual orientation [2, 11], and HIV serostatus [10, 12, 13]). Black/African Americans living with HIV also have fewer social resources available to cope with these forms of oppression, including fewer social engagements and fewer social network ties [9, 11, 14]). Moreover, compared to HIV-negative adults, people living with HIV (PLWH) in general report higher levels of loneliness [15, 16], which is defined as the distress that exists between actual and desired relationships (which is different from the concept of living alone or aloneness). Current rates of loneliness among PLWH have been reported to range from 30 to 60% [15, 17, 18].

Loneliness is associated with adverse health outcomes in the general population [19–21], including substance use and abuse, poor diet, physical inactivity, poor general health, and increased mortality rates, as well as having multiple sexual partners [22–26]. Furthermore, PLWH who report loneliness are more likely to use alcohol, use tobacco, report depression, have poor sleep, have fewer relationships, and be non-adherent to ART—all of which impact their overall physical and mental health [15, 27, 28]. Studies have also shown that loneliness is associated with pronounced changes in the neuroendocrine and immune functions, thus acting as a moderator of health through high rates of inflammation, poorer immune responses, higher levels of viral activity, and stress-induced cortisol dysregulation [29–32]. Higher risk of comorbidities and lower quality of life has also been found among PLWH who report being lonely [33].

To our knowledge, only a few studies have investigated correlates of loneliness among PLWH and even fewer among PLWH who are Black/African American [15, 27, 34, 35]. The current study sought to address this gap by further understanding (a) the sociodemographic and social determinant characteristics of Black adults living with HIV who are lonely and (b) the implications of loneliness for their health, including their mental health, physical health, and ART adherence.

## Methods

### Study Population and Design

This study used baseline data from an ongoing randomized controlled trial of an intervention to improve viral suppression, ART adherence, and retention in care in Black adults living with HIV (#NCT03331978) [36]. Participants (*n* = 304) were recruited through a community-based HIV service organization in Los Angeles County, CA, USA, via flyers; provider referrals; radio shows; online and print advertisements; online promotions; outreach to staff and clients of relevant community organizations, at community events, and on the streets; and outreach onsite (at the community partner organization). Recruitment and enrollment occurred from January 2018 to July 2020.

Inclusion criteria were (1) 18 years of age or older, (2) Black/African American racial/ethnic identity, (3) able to communicate in English (either written or spoken), (4) HIV-positive serostatus, (5) prescribed ART for the first time at least 6 months ago, (6) self-reported adherence problems (i.e., missed at least one ART dose in the past month) and/or detectable viral load, and (7) willingness to use an electronic adherence monitoring device. All participants consented prior to participating in the study and received $30 for the baseline assessment and $30 for a 1-month follow-up visit. This study was approved by the RAND Human Subjects Protection Committee (HSPC 2016-0940).

### Measures

#### Sociodemographic Characteristics

We assessed age (continuous), gender, gender identity (coded as cisgender male, cisgender female, and transgender, gender queer, or gender non-conforming), sexual orientation (coded as heterosexual vs. gay, lesbian bisexual, something else, or “other”), marital status (coded as married/living with significant other or not married/living with significant other), education (coded as less than high school graduate vs. high school graduate), income (< $10,000 vs. $10,000 or greater), housing status in the last 12 months [coded as stable (e.g., own or rent) vs. unstable (e.g., homeless)], employment status (coded as full-time or part-time work vs. not working), and years since HIV diagnosis.

#### Social Determinants of Health Characteristics

An unmet needs measure was adapted from items from the HIV Cost and Services Utilization Study to assess needs for financial/benefits, housing, mental health, food, transportation, and substance use treatment [37, 38]. For each of 10 types of services, participants were asked whether they: (a) needed the service in the past 6 months and, if so, (b) whether they received the service. We operationalized unmet needs as the sum of types of services which were needed but not received (range 0–10). Participants were also asked to report any adult incarceration history (yes/no).

Discrimination was assessed with the Multiple Discrimination Scale (MDS), which has separate subscales (each enquiring about the presence of 10 discrimination events such as being denied or losing a job, being insulted, or physically assaulted) for discrimination related to HIV serostatus (MDS-HIV; alpha = .86), race/ethnicity (MDS-Race; alpha = .90), and sexual orientation (MDS-Gay; alpha = .92) [39]. The MDS response options were yes and no for each item; the sum of each subscale was computed representing the number of discrimination events in the past year.

Internalized HIV stigma was assessed with the Internalized AIDS-Related Stigma Scale (alpha = .88) [40]. Disclosure of HIV serostatus was assessed separately for friends, family, or partners (with response options “some,” “none,” or “all”); items were combined into one dichotomous measure (“no” versus “any” disclosure). Participants were also asked to identify their level of social support in the past 4 weeks via a set of 5 questions (e.g., “How often did you have someone to love and make you feel wanted?”) with response options ranging from 1 = “none of the time” to 5 = “all of the time” (alpha = .91) [41]. The Drug Abuse Screen Test (DAST-10) was used to measure problematic substance use; for analysis, the score was dichotomized at 0–4 representing low problem level and scores of 5–10 representing substantial to severe drug abuse problems [42, 43].

#### Loneliness

Loneliness was assessed as the mean of eight items from the revised UCLA Loneliness Scale, with answer choices of never (1) to often (4) (alpha = .82) [44].

#### Health Outcomes

##### Physical and Mental Health

Two domains from the patient-reported outcomes measurement information system (PROMIS) global health item set were used to assess physical health and mental health [45]. Participants were asked to self-rate their physical health and mental health on a 5-item Likert scale ranging from excellent to poor.

##### Adherence

Adherence was monitored daily after baseline for one month using the medication event monitoring system (MEMS; AARDEX, Inc.), which records bottle opening dates and times. Participants received the cap when they completed the baseline survey; the present analysis included MEMS data from the baseline survey until 1 month after the baseline survey, when MEMS data were downloaded (prior to randomization for the clinical trial). Adherence was operationalized as a continuous measure of percentage of prescribed doses taken. The adherence data was adjusted using response to a brief survey collected at the same time as MEMS data to assess when the cap was not used as intended (e.g., how often a bottle was opened without removing a dose) [36, 46].

##### Depression

Depression was measured by the eight-item Patient Health Questionnaire depression scale (PHQ-8) that is established as a valid diagnostic and severity measure for depressive disorders [47]. Each of the eight items were scored between 0 to 3, providing a 0 to 24 severity score. The scores were then dichotomized with overall scores of ≤ 10 indicating mild depression severity and those > 10 indicating moderately severe to severe scores necessitating treatment plans.

### Statistical Analysis

For descriptive purposes, we generated means with standard deviations and frequencies as appropriate for all sociodemographic and social determinant characteristics. Bivariate linear regression models were conducted with loneliness as the dependent variable and independent variables including sociodemographic characteristics, social determinants of health factors, and health outcomes (general physical health, general mental health, adherence, and depression). Finally, adjusted linear regression analysis was conducted predicting each health outcome with loneliness, controlling for any sociodemographic variables that were found to be significantly associated (*P* < 0.05) with both loneliness and the health outcome.

Because MEMS data were not available for all participants, non-response weights were derived to account for any bias that may be introduced by excluding participants without MEMS data. Weights were developed as the inverse of the predicted probability of having MEMS baseline data from a logistic regression model including a variety of baseline characteristics. All analyses involving MEMS data used these weights; all other analyses are unweighted.

## Results

Among the 304 participants, two participants did not complete the loneliness scale, and thus, the present analysis was conducted for 302 individuals. Of those, 95% reported symptoms of loneliness with 35% reporting mild loneliness, almost half (48%) reporting moderate loneliness, and 12% reporting severe loneliness. The mean loneliness score (with possible range 1–4) was 2.27 (SD = 0.71), which is consistent with moderate symptoms.

### Sociodemographic Characteristics

Sociodemographic characteristics and their bivariate associations with loneliness are presented in Table 1. Over half of the population (51%) were 50 years and above, and the mean age was approximately 48 years old (SD = 12.5); 19% identified as cisgender female. Approximately 26% identified as straight or heterosexual, 11% were married/living with a partner, 15% had less than a high school education, and 16% were working full time or part time. The mean time since HIV diagnosis was 17 years, and half had an annual income of $10,000, and about half had stable housing.

**Table 1:** Characteristics of a sample of Black adults living with HIV (*n* = 302)

	*M* (SD), range or %
Sociodemographic Characteristics	
Age *M* (*SD*), range	47.7 (12.5), 18–75
Straight or heterosexual sexual orientation	26.2%
Cisgender female identity	18.9%
Married/living with significant other	11.0%
Less than high school education	14.6%
Working full time or part time	15.6%
Income < $10,000	50.2%
Stable housing (e.g., own or rent)	48.2%
Time (years) since HIV diagnosis *M* (*SD*), range	16.7 (9.7), 0.4–39.2
Social Determinants of Health Factors	
Any incarceration history	51.0%
Internalized HIV stigma *M* (*SD*), range	2.8 (1.2), 1–5
Disclosed HIV to any friends/family/partners	95.3%
Unmet needs *M* (*SD*), range	1.2 (1.5), 0–8
Discrimination related to HIV serostatus	1.5 (2.3), 0–10
Discrimination related to race	2.1 (2.9), 0–10
Discrimination related to sexual orientation	1.6 (2.8), 0–10
Social support	2.6 (1.3), 1–5
Drug abuse screening test (DAST) score > 5	26.8%
Health Outcomes	
Adherence (percentage of prescribed doses taken in past month) *M* (SD)	60.1 (49.1), 0–100
Physical health score *M* (SD)	3.2 (0.8), 1–5
Mental health score *M* (SD)	2.7 (0.9), 1–5
Depression (PHQ8 score ≥ 10)	7.3 (6.5), 0–24

Physical health and mental health were assessed with the PROMIS global health item set and depression was assessed with the PHQ8

In bivariate regression models (see Table 2), partner status and housing status were associated with loneliness: Those who were married or living with a partner experienced lower rates of loneliness [*b*(SE) = − 0.32 (0.13), *P* = 0.02] than those who were not married or living with a partner. Those with stable housing experienced lower levels of loneliness than those in unstable housing [*b*(SE) = − 0.20 (0.08), *P* = 0.02]. Time since HIV diagnosis had a marginally negative correlation with loneliness.

**Table 2:** Bivariate associations with loneliness

	*Bivariate association with loneliness*	
	b (SE)	*P* value
Sociodemographic characteristics		
Age *M* (*SD*), range	− 0.01 (< 0.01)	0.14
Straight or heterosexual sexual orientation	− 0.14 (0.09)	0.15
Cisgender female identity	− 0.08 (0.11)	0.47
Married/living with significant other	− 0.32 (0.13)	**0.02**
Less than high school education	0.09 (0.10)	0.38
Working full time or part time	− 0.09 (0.13)	0.45
Income < $10,000	0.15 (0.08)	0.07
Stable housing (e.g., own or rent)	− 0.20 (0.08)	**0.02**
Time (years) since HIV diagnosis *M* (*SD*), range	− 0.01 (< 0.01)	0.06
Social determinants of health factors		
Any incarceration history	0.07 (0.08)	0.42
Internalized HIV stigma *M* (*SD*), range	0.28 (0.03)	**< 0.001**
Disclosed HIV to any friends/family/partners	0.08 (0.13)	0.54
Unmet needs *M* (*SD*), range	0.13 (0.03)	**< 0.001**
Discrimination related to HIV serostatus	0.12 (0.02)	**< 0.001**
Discrimination related to race	0.09 (0.01)	**< 0.001**
Discrimination related to sexual orientation	0.08 (0.01)	**< 0.001**
Social support	− 0.23 (0.03)	**<0.001**
Drug abuse screening test (DAST) score >5	0.26 (0.10)	**0.009**

The bolded items show statistical significance at *p* < 0.05

### Social Determinants of Health

In our sample, approximately half (51%) had a history of incarceration, 27% reported substantial to severe drug abuse, and nearly all (95%) had disclosed HIV to at least some friends/family/partners. Participants reported few unmet needs (*M* = 1.2; SD = 1.5; range = 0–8). The most common unmet needs were housing (27%), transportation assistance (19%), employment assistance (18%), and mental health treatment (15%).

There were low levels of past-year discrimination due to HIV status (*M* = 1.5; SD = 2.3; range = 0–10), race (*M* = 2.1; SD = 2.9; range = 0–10), and sexual orientation (*M* = 1.6; SD = 2.8; range = 0–10), while levels of internalized HIV stigma (*M* = 2.8; SD = 1.2; range = 1–5) and social support (*M* = 2.6; SD = 1.3; range = 1–5) were moderate.

In bivariate linear regression models (see Table 2), those with substantial to severe drug abuse problems [*b*(SE) = 0.26 (0.10), *P* = 0.009] and those with higher levels of internalized HIV stigma [*b*(SE) = 0.28 (0.03), *P* < 0.001], depression [*b*(SE) = 0.74 (0.07), *P* < 0.001], unmet needs [*b*(SE) = 0.13(0.03), *P* < 0.001], discrimination related to HIV serostatus [*b*(SE) = 0.12 (0.02), *P* < 0.001], race [*b*(SE) = 0.09 (0.01), *P* < 0.001], and discrimination related to sexual orientation [*b*(SE) = 0.08 (0.01), *P* < 0.001] had higher loneliness scores. In contrast, those with higher social support experienced lower levels of loneliness [*b*(SE) = − 0.23 (0.03), *P* < 0.001].

### Health Outcomes

Average adherence in the sample was 60.1% (SD = 49.1%) of prescribed doses taken in the month following baseline, and approximately 60% of the sample achieved at least 75% adherence. Most participants rated their mental health as average (*M* = 2.78; SD = 0.91; range 1–5) and their physical health as above average (*M* = 3.22; SD = 0.82; range 1–5). Participants reported average levels of depression (*M* = 7.3; SD = 6.5; range 0–24).

Stable housing was significantly associated with both loneliness and depression and thus was used as a covariate in the multivariable regression model predicting depression. In regressions modeling health outcomes (see Table 3), loneliness was found to be a significant predictor of worse physical health [*b*(SE) = − 0.37 (0.06), *P* < 0.001], worse mental health [*b*(SE) = − 0.72 (0.05), *P* < 0.001], and greater depression [*b*(SE) = 5.49 (0.41), *P* < 0.001] and was marginally associated with lower adherence [*b*(SE) = − 3.96 (2.26), *P* = 0.08].

**Table 3:** Multivariable regressions predicting health outcomes with loneliness in a sample of black adults living with HIV

Health outcomes	*n*	*b* (SE)	*P* value
Adherence (percentage of prescribed doses taken in past month)^	233	− 3.96 (2.26)	0.08
Physical health score	302	− 0.37 (0.06)	< 0.001
Mental health score	302	− 0.72 (0.05)	< 0.001
Depression*	300	5.49 (0.41)	< 0.001

^Results employ nonresponse weights to account for presence of adherence data

*Results adjusted with stable housing

## Discussion

The present study sought to address gaps in research on loneliness among Black adults living with HIV by further understanding (a) the sociodemographic and social determinant characteristics of Black adults living with HIV who are lonely and (b) the implications of loneliness for their health. Loneliness was very common in this sample and associated with several sociodemographics and social determinants, including stigma and multiple forms of discrimination, and was associated with poor physical and mental health.

Internalized HIV stigma and multiple forms of discrimination were among the strongest correlates of loneliness. Black persons living with HIV are often marginalized due to their intersecting stigmatized identities (being Black, HIV-positive, and a sexual minority). As has been noted by other researchers, Black, HIV-positive, and non-heterosexual individuals are triply stigmatized and therefore carry worse outcomes [48, 49]. Intersectionality theory emphasizes the importance of placing a person’s experiences in the context of their multiple interconnected identities and forms of oppression that they face [50–52]. Thus, we do not know if the rate of internalized HIV stigma seen in our sample is solely related to their HIV status and experience, or due to their interconnected identities influenced by existing race and/or sexual orientation internalized stigma. What is clear, however, is that loneliness is greater among individuals with internalized HIV stigma and among those who experience discrimination related to their HIV status, race, and sexual orientation.

While the level of unmet needs in our sample was lower than in most reported studies [53–56], we found that those with unmet needs experience loneliness more than those who do not. Neurobiology research on loneliness posits that lonely people have less modular connections between attention, visual, and “default mode networks” in the brain, which are signals that a social need is unfulfilled [57, 58]. Thus, when needs are unmet in an individual’s life, their brain patterns may be impacted in the areas associated with loneliness. While such research is very new and limited, neurobiology argues that loneliness signals that basic needs are not met [57]. In Black communities, the longevity of needs being unmet can exacerbate the loneliness felt and lead to more serious consequences for physical and mental health [59–61].

Loneliness was found to be a significant correlate of physical health, mental health, and depression. Loneliness has been shown to shift the immune system responsivity, thereby impairing one’s physical health [62, 63], and the neurochemistry of the brain, thus impacting mental health [58, 64]. Consistent with this prior research, in our sample that evidenced high rates of loneliness, we saw corresponding reports of poor physical and mental health. Regarding depression, there is a growing body of evidence showing that loneliness is a proximal factor preceding depression that has a significant effect even at moderate levels on depression [65–67]. In accordance with the majority of our sample having moderate levels of loneliness, depression was common as well.

This study also identified positive protective factors for loneliness in our sample. Consistent with well-known associations between loneliness and sociodemographic characteristics found in literature, our study revealed that being married or cohabitating with a partner and having stable housing were negatively associated with loneliness [35, 68–70]. In addition, those with higher social support experienced less loneliness than those with less social support. Social support is considered to be the most important factor in alleviating loneliness and its effects and may even predict the trajectory of loneliness [71–73]. Among PLWH, social support through family and friends, clinical staff, and peer volunteers aids them in navigating their health and adherence to treatment [74]. Therefore, it is important to encourage support in the social networks of PLWH and to further explore social support characteristics, in order to identify which types of support (e.g., emotional, tangible, mental, caregiving) [75–77] are most beneficial. Future studies can use randomized controlled trial designs to test effects of referring clients to support groups with similar peers on decreasing feelings of loneliness and, in so doing, improving overall health and well-being [78, 79].

The results of this study should be viewed in light of several limitations. First, we recruited a convenience sample in Los Angeles County, California, with socioeconomic disadvantages such as low income and unstable housing. Thus, the results may not be generalizable to all Black adults living with HIV in the USA. Due to the small subsample sizes of heterosexual participants, women, and transgender individuals, subgroup analyses were not conducted; future studies should examine these subgroups in better distributed samples. Future studies should also explore loneliness in rural and less-resourced populations, as this study was conducted in a well-resourced urban setting and a large community-based organization connected to a federally qualified health center. In addition, with the exception of the adherence measure, analyses were conducted cross-sectionally with observational data, and therefore, we cannot infer a causal direction of the associations. Further, we did not assess whether participants experienced loneliness pre-HIV diagnosis or whether they had lost social network members with HIV prior to the advent of highly active antiretroviral therapy (HAART) as a result—both of which may have been correlated with their present-day perceptions of loneliness.

Despite the limitations, our findings are an important first step in understanding loneliness among Black adults living with HIV. Given the limited prior research, this study provides valuable information that may help researchers better understand the characteristics of loneliness among Black adults with HIV and its impact on health. Our findings suggest that while loneliness is a growing public health problem among all racial and ethnic groups, Black adults living with HIV, who experience multiple intersectional stigmas, may have higher rates of loneliness than other populations and require targeted interventions and resources.
